# Effects of Incretin-Based Pharmacotherapies on Lean Body Mass in Adults With Overweight, Obesity, or Type 2 Diabetes: A Systematic Review With Prespecified Network Meta-Analysis Feasibility Assessment

**DOI:** 10.7759/cureus.117738

**Published:** 2026-10-10

**Authors:** Zahi M Alhamad, Naif AlShalhoub, Abdulrahman Al-mana, Faisal E Almutairi, Hamad M Alshalhoub

**Affiliations:** 1 Department of Family Medicine, Prince Sultan Military Medical City, Riyadh, SAU; 2 Department of Family Medicine, Royal Guard Medical Services, Riyadh, SAU; 3 College of Medicine, Ibn Sina National College for Medical Studies, Jeddah, SAU

**Keywords:** body composition, dxa, glp-1 receptor agonists, incretin therapy, lean body mass, network meta-analysis, obesity, semaglutide, tirzepatide, weight loss

## Abstract

Incretin-based pharmacotherapies produce substantial weight loss, but their comparative effects on lean tissue remain incompletely characterized. We systematically reviewed randomized controlled trials in adults with overweight, obesity, and/or type 2 diabetes. The prospectively registered primary outcome was the between-group difference in relative percentage change from baseline in total lean body mass measured by dual-energy X-ray absorptiometry (DXA) at 48 to <80 weeks; absolute DXA change in kilograms was the key secondary outcome. The auditable record-level dataset comprised 6,526 imported records from PubMed and ClinicalTrials.gov; after the removal of 250 within-PubMed duplicates, 6,276 records were screened, and 52 trial families met review-level eligibility. Three direct contrasts had defensible precision for the registered primary outcome: pooled tirzepatide versus placebo, -8.3 percentage points (95% confidence interval {CI}: -10.6 to -6.1); semaglutide 1.0 mg versus placebo, -3.81 (-6.57 to -1.06); and semaglutide 2.4 mg versus placebo, -6.06 (-8.79 to -3.33). Because the two BELIEVE semaglutide contrasts shared a placebo group and their joint covariance was unavailable, they were not treated as independent edges in a single dose-specific network meta-analysis (NMA). Separate placebo-anchored sensitivity comparisons of pooled tirzepatide versus semaglutide were -4.49 percentage points (95% CI: -8.05 to -0.93) for semaglutide 1.0 mg and -2.24 (-5.78 to 1.30) for semaglutide 2.4 mg; both were graded very low confidence. For the key secondary absolute DXA outcome, mean differences versus placebo were -3.43 kg (95% CI: -4.74 to -2.13) for semaglutide 2.4 mg, -4.40 kg (-5.60 to -3.20) for pooled tirzepatide, and -5.90 kg (-7.28 to -4.52) for mazdutide 9 mg. DXA-derived lean mass is not synonymous with skeletal-muscle mass or function, and these reductions should not by themselves be interpreted as evidence of sarcopenia or clinically harmful muscle loss. Randomized evidence therefore supports measurable lean-mass loss during incretin-associated weight reduction, but sparse network geometry, heterogeneous estimands, selected body-composition substudies, and incomplete reporting preclude a reliable comparative treatment ranking.

## Introduction and background

Obesity and type 2 diabetes impose a substantial and increasing global disease burden. In 2022, approximately 2.5 billion adults worldwide were overweight, including more than 890 million living with obesity; 43% of adults were overweight, and 16% were living with obesity [1]. The International Diabetes Federation estimated that 589 million adults aged 20-79 years were living with diabetes in 2024, with diabetes accounting for more than 3.4 million deaths annually and global diabetes-related health expenditure exceeding US$1 trillion [2]. Against this background, the rapidly expanding use of incretin-based therapies has increased interest in the composition, rather than only the magnitude, of treatment-associated weight loss [3,4]. Incretin-based therapies, including glucagon-like peptide-1 (GLP-1) receptor agonists and newer dual- and triple-receptor agonists, can produce substantially greater weight loss than was previously typical with pharmacotherapy. As these agents are used more widely and for longer periods, a clinically important question is whether reductions in fat mass are accompanied by the loss of lean tissue.

Interpretation requires a clear distinction between body-composition and muscle-function outcomes. Dual-energy X-ray absorptiometry (DXA)-derived lean body mass includes not only skeletal muscle but also water and other non-fat soft tissues; it is not synonymous with anatomically measured skeletal-muscle mass, muscle quality, strength, or physical performance. A reduction in DXA-derived lean mass therefore does not by itself establish sarcopenia, clinically harmful muscle loss, or functional deterioration. Its clinical significance may differ substantially according to age, baseline frailty or muscle reserve, comorbidity, nutritional intake, resistance exercise, and functional reserve [5].

The literature is also methodologically heterogeneous. Trials have reported lean mass in kilograms, percentage change from baseline, lean mass as a proportion of body weight, fat-free mass, lean soft tissue, appendicular lean mass, and magnetic resonance imaging (MRI)-derived regional or whole-body tissue volumes. These outcomes answer related but non-equivalent questions. In this review, the estimand refers to the specific treatment effect defined by outcome construct, analysis population, treatment comparison, time point, and the handling of intercurrent events. Transitivity refers to whether important effect modifiers are sufficiently comparable across randomized comparisons to justify indirect treatment comparisons. Network meta-analysis (NMA) feasibility therefore requires not only a connected graph but also compatible outcomes, time windows, estimands, and defensible uncertainty.

We therefore aimed to compare the effects of eligible incretin-based pharmacotherapies on total lean body mass while preserving the prespecified hierarchy of outcomes and measurement methods. The registered primary estimand was the between-group difference in percentage change from baseline in total DXA lean body mass at 48 to <80 weeks, with absolute DXA change in kilograms as the key secondary outcome. The primary NMA was conditional on the existence of a connected network of valid randomized contrasts; if that condition was not met, the available direct primary evidence was reported without replacing the registered endpoint.

## Review

Methods

Protocol and Registration

The review was conducted according to a prespecified protocol (version 4.1), PRISMA 2020 guidance, and International Prospective Register of Systematic Reviews (PROSPERO) registration CRD420261483502 [6]. The PROSPERO record was registered on 20 August 2026 and amended on 23 August 2026 to document limitations in database access. Eligibility criteria, outcome definitions, time windows, risk-of-bias methods, and planned statistical analyses were not changed. A numerical data lock preserved an auditable analysis set while allowing later author-supplied information to be incorporated through dated, study-specific amendments.

AI-Assisted Workflow Disclosure

AI-assisted tools were used under author supervision for literature organization, search-term refinement, preliminary screening support, structuring extraction and audit tables, deterministic coding/calculation checks, consistency checking, and language editing. No AI-generated eligibility decision, numerical value, Risk of Bias 2 (RoB 2) judgment, statistical conclusion, or final interpretation was accepted without verification against the underlying source and author review. AI tools were not treated as independent reviewers or authors (Table 1).

**Table 1:** Prespecified eligibility criteria and outcome hierarchy DXA, dual-energy X-ray absorptiometry; MRI, magnetic resonance imaging; CT, computed tomography; BIA, bioelectrical impedance analysis

Domain	Operational criterion
Population	Adults ≥18 years with overweight, obesity, and/or type 2 diabetes. Overweight/obesity could independently establish eligibility even when another diabetes phenotype or disease context was present
Design	Randomized controlled trials. Randomized body-composition substudies were eligible when original randomization was preserved and subset selection was not a post-randomization response-based selection
Intervention	Eligible incretin-based pharmacotherapy according to the registered mechanism definition; combination regimens containing non-target active agents were not allowed to create eligible monotherapy nodes
Comparator	Placebo, usual care, or eligible incretin comparator. Non-target active comparators did not serve as primary-network bridge nodes
Minimum duration	≥12 weeks. Windows: 12 to <24, 24 to <48, 48 to <80 (primary), and ≥80 weeks
Primary modality/outcome	DXA-derived total lean body mass; percentage change from baseline
Key secondary	Absolute change in total DXA lean body mass (kg)
Separate outcome families	BIA-derived lean measures, appendicular lean mass, fat-free mass/lean soft tissue, and MRI/CT regional or whole-body lean-volume outcomes were not pooled as equivalent to total DXA lean body mass

Outcomes and Estimand Definitions

The registered primary effect measure was the between-group mean difference in percentage change from baseline in total DXA lean body mass, expressed in percentage points. The key secondary effect measure was the between-group mean difference in absolute change (kg). When a trial reported both treatment-regimen/treatment-policy and efficacy/on-treatment estimands, the randomized treatment-regimen/treatment-policy-like estimand was preferred where available; alternative estimands were retained for sensitivity analysis and indirectness assessment.

A percentage of body weight that is lean mass, or the fraction of total weight loss attributable to lean tissue, was not treated as the registered percentage-change outcome. Trial-level eligibility was broader than quantitative pooling: a trial could be eligible if it contained a protocol-eligible body-composition outcome, whereas pooling required compatibility in outcome construct, measurement modality, unit/estimand, time window, analysis population, and uncertainty. This distinction prevented non-equivalent body-composition outcomes from being used merely to complete a network.

Information Sources and Search Strategy

PubMed/Medical Literature Analysis and Retrieval System Online (MEDLINE) was searched on 24 August 2026 using two complementary routes: a broad randomized trial strategy and an outcome-sensitive body-composition strategy. Search concepts combined incretin/GLP-1 receptor agonist and dual/triple agonist terms and individual drug names with obesity/overweight/type 2 diabetes terms, randomized trial terms, and a body-composition/muscle concept that included lean body mass, lean mass, fat-free mass, lean soft tissue, appendicular lean mass, skeletal muscle, body composition, DXA/DEXA, bioimpedance, MRI, computed tomography (CT), muscle strength, handgrip, and physical performance. The two PubMed searches yielded 6,368 records before within-PubMed deduplication; the removal of 250 duplicates left 6,118 unique PubMed records. ClinicalTrials.gov was exported on the same date and contributed 158 unique registry records.

Cochrane Central Register of Controlled Trials (CENTRAL) was searched separately using the prespecified concepts and returned 8,250 trial records. A reproducible citation export could not be obtained from the interface available to the review team, so those records could not be imported into record-level deduplication and screening and were not individually screened. Embase, Scopus, and Web of Science were not searched because institutional/subscription access was unavailable; the WHO International Clinical Trials Registry Platform (ICTRP) did not provide a reproducible native export during the final search window. To mitigate retrieval limitations, we used ClinicalTrials.gov, backward and forward citation checking where feasible, trial-family tracing, cross-checking against recent systematic reviews, targeted evidence updates, and direct investigator/sponsor contact for high-priority unresolved trials. The implications of incomplete bibliographic coverage were evaluated explicitly rather than treated as equivalent to a complete multi-database search.

Study Selection and Trial-Family Reconciliation

The auditable record-level flow comprised 6,526 records imported from PubMed and ClinicalTrials.gov. After the removal of 250 within-PubMed duplicates, 6,276 records were screened. Trial reports were linked to parent studies using registration identifiers, interventions, populations, dates, and parent-substudy relationships to minimize double counting. Following screening, citation checking, registry review, and trial-family reconciliation, 117 reports or registry records underwent final eligibility adjudication. Of these, 62 were excluded, and two were retained as ongoing or monitoring records. The remaining 53 included reports or registry records representing 52 unique randomized trial families. These 53 records comprised 48 original publications, two linked reports of already-counted trial families, and three additional registry records. Because one publication reported two distinct randomized trials (LEAD-2 and LEAD-3), the 48 original publications represented 49 publication-based trial families; together with three registry-only trial families, this yielded 52 unique trial families.

Screening and evidence adjudication were conducted by members of the review team using prespecified criteria and structured cross-checking. The retained audit trail does not establish a fully independent duplicate screening pass for every one of the 6,276 records; this is reported transparently as a review limitation rather than retrospectively described as universal duplicate screening. Uncertain eligibility decisions were resolved by source re-verification and team adjudication.

Data Extraction and Source Hierarchy

Numerical data were prioritized in the following order: primary article tables or text, official supplementary files or source-data spreadsheets, regulatory or registry records reporting trial-derived statistics, and secondary reviews for the corroboration or identification of unresolved values. Numerical values from secondary sources were not used in the principal synthesis unless they could be verified against the original trial family. Quantitative fields entering weighted analyses underwent duplicate extraction or an independent numerical cross-check against the source. Disagreements or uncertain fields were resolved by source re-verification and team discussion; unresolved values were retained as data-blocked rather than inferred from non-equivalent outcomes.

When necessary, SDs or standard errors (SEs) were derived from reported confidence intervals (CI) or other exact statistics only when the derivation referred to the same estimand and was mathematically valid. We did not derive change-score variance from baseline and follow-up SDs without a defensible within-person correlation, and we did not assume that adjusted least-squares means from the same analysis of covariance (ANCOVA) or mixed model were independent. Twenty targeted investigator/sponsor requests were logged by 24 August 2026. Returned data were incorporated only when the randomized comparison, eligible outcome, time point, analysis population, and uncertainty provenance were clear.

Risk of Bias

Risk of bias was assessed at the outcome level using the Cochrane Risk of Bias 2 (RoB 2) tool, covering the domains of the randomization process, deviations from intended interventions, missing outcome data, the measurement of the outcome, and the selection of the reported result [7]. All included trial families were considered for outcome-level assessment using an eligible lean- or muscle-related body-composition result. When a sufficiently reported outcome-level result was available, domain-level and overall RoB 2 judgments were assigned. Trial families in which an eligible outcome was identified but no sufficiently reported result was available were classified as not assessable (NR) rather than assigned an unsupported risk-of-bias judgment. Assessments incorporated primary reports, supplementary materials, protocols, registry information, and author-supplied data where available, with uncertain judgments resolved by source re-verification and study-team adjudication. The SURPASS J-mono body-composition outcome was assessed using the same framework; the underlying randomized substudy is reported by Yabe et al. [8].

Statistical Analysis and Network Feasibility

Treatment nodes were defined by randomized agent and dose whenever data permitted. If a body-composition analysis pooled randomized doses, the pooled agent-level contrast was retained rather than reconstructing dose-specific effects. Multi-arm trials with a shared control were not treated as independent two-arm studies. A contrast entered weighted synthesis only when both the treatment effect and uncertainty were reported or could be derived without unverifiable assumptions. The prespecified plan was random-effects pairwise meta-analysis and frequentist random-effects NMA when connectivity, transitivity, and consistency were credible. In the final primary-window evidence, those conditions were not met for a joint dose-specific model; a fixed-effect model would not solve the shared-control covariance problem.

For a reported two-sided 95% confidence interval, SE was reconstructed as (upper limit - lower limit)/(2 × 1.96). For exploratory placebo-anchored indirect comparisons derived from independent trials, MD(A vs B) = MD(A vs placebo) - MD(B vs placebo), Var(indirect) = SE(A vs placebo)^2^ + SE(B vs placebo)^2^, and the 95% CI was estimate ± 1.96 × SE. These formulas were not used to treat the two BELIEVE semaglutide comparisons as mutually independent because both share the same placebo group.

After the BELIEVE amendment, three direct contrasts had defensible precision for the registered relative percentage DXA outcome. The two BELIEVE semaglutide contrasts are correlated because they share the same placebo participants. Treating them as independent observations in one joint model would double-count shared-control information and could narrow uncertainty. Because the covariance between the two adjusted contrasts was unavailable, no correlation coefficient was imputed. Instead, each BELIEVE contrast was retained as valid direct evidence and compared separately with the independent SURMOUNT-1 placebo contrast in two dose-specific placebo-anchored sensitivity analyses. Each individual sensitivity comparison therefore avoids the unknown within-BELIEVE covariance; the two resulting indirect estimates should nevertheless not be interpreted as independent of one another when viewed jointly.

The key secondary absolute DXA evidence formed a placebo-centered star with one study per core active-treatment edge and no closed loops. Consequently, edge-specific between-study heterogeneity and network incoherence were not empirically estimable; they were reported as not estimable rather than assumed to be zero. The short-duration two-study liraglutide pool was reported descriptively using fixed-effect inverse-variance weighting; with k=2, I² and any tau² estimate are unstable and do not establish clinical homogeneity. All SE reconstructions and indirect-comparison confidence intervals were computationally rechecked during revision using Python 3.13.5 (Python Software Foundation, Fredericksburg, VA), NumPy 2.3.5, and pandas 2.2.3.

Certainty of Evidence

Confidence was assessed using the Confidence in Network Meta-Analysis (CINeMA) framework, considering domains informed by outcome-level RoB 2, reporting bias, indirectness/transitivity, imprecision, heterogeneity, and incoherence. The registered primary dose-specific network did not receive a formal network-wide grade because the simultaneous inclusion of both BELIEVE dose contrasts would require their unavailable covariance. For one-study edges, heterogeneity was not estimable; with no closed loops, incoherence was not estimable. Because no validated minimally important difference exists for total lean body mass, imprecision was judged from confidence-interval width, whether intervals crossed the null, and the stability of effect direction, while clinical magnitude was interpreted separately.

Results

Screening and Quantitative Readiness

The auditable record-level flow included 6,526 imported records from PubMed/MEDLINE and ClinicalTrials.gov. After the removal of 250 within-PubMed duplicate records, 6,276 records were screened, of which 117 reports or registry records proceeded to final eligibility adjudication. Sixty-two reports or registry records were excluded, and two ongoing studies were retained for monitoring. The remaining 53 included reports or registry records represented 52 unique randomized trial families after trial-family reconciliation. CENTRAL was searched separately and returned 8,250 records; however, because a reproducible citation export could not be obtained, these records were not imported into the auditable record-level screening workflow or individually screened. The recruiting COMPARE-AT study was retained for future-update monitoring and did not contribute quantitative data (Figure 1) [9].

**Figure 1:**
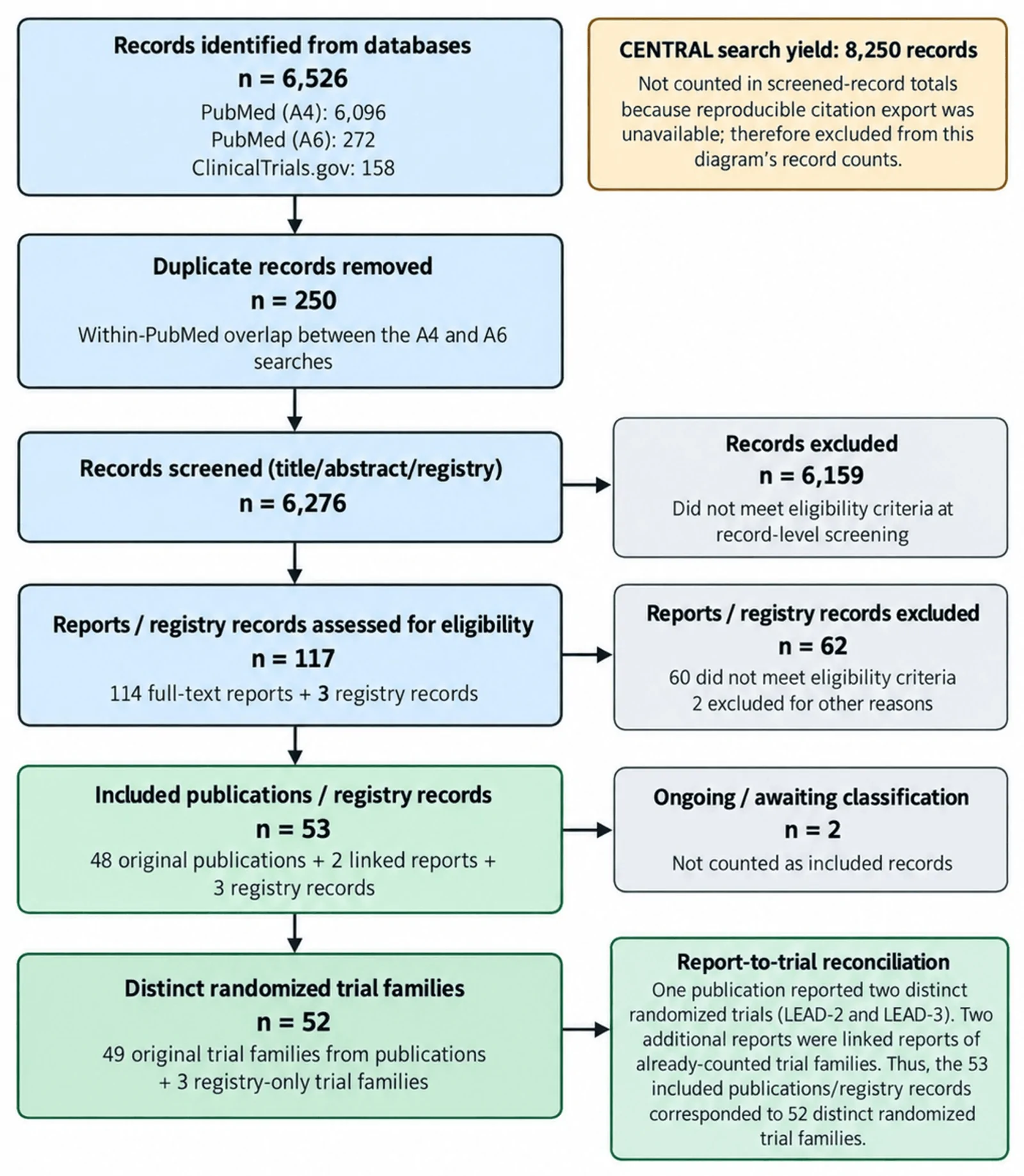
PRISMA flow diagram of study identification, screening, and inclusion Study selection and trial-family reconciliation. After screening and final eligibility adjudication, 53 included reports or registry records represented 52 unique randomized trial families. Two records were linked reports of already-counted trial families, while one publication reported two distinct randomized trials, LEAD-2 and LEAD-3. Two ongoing or monitoring records were not counted among the included studies; the recruiting COMPARE-AT trial was retained for future-update monitoring [9]. CENTRAL yielded 8,250 records, but these could not be exported reproducibly for auditable record-level screening and were therefore not included in the screened-record totals shown CENTRAL: Cochrane Central Register of Controlled Trials

Characteristics of Included Studies

The included evidence comprised 52 randomized trial families with protocol-eligible lean- or muscle-related body-composition outcomes. Study populations included adults with overweight or obesity, type 2 diabetes, polycystic ovary syndrome, prior bariatric surgery, HIV-associated lipohypertrophy, older age, and other selected clinical contexts. Body-composition assessment included DXA, bioimpedance-based methods, MRI/CT-derived lean-tissue measures, and other prespecified modalities. Eligibility for the review did not imply eligibility for quantitative synthesis; studies were synthesized only when outcome construct, measurement modality, time window, estimand, analysis population, and treatment-effect uncertainty were sufficiently compatible (Table 2).

**Table 2:** Characteristics of the 52 included randomized trial families with eligible lean- or muscle-related body-composition outcomes Reference counts do not correspond one-to-one with trial-family counts. Some trial families are supported by more than one report, whereas LEAD-2 and LEAD-3 are two distinct randomized trials reported in a single shared publication. Accordingly, the 52 trial-family rows are supported by multiple bibliographic references, with some trial families supported by more than one report and LEAD-2 and LEAD-3 representing two distinct randomized trials reported in a single shared publication BIA, bioelectrical impedance analysis; BIS, bioimpedance spectroscopy; CT, computed tomography; DXA/DEXA, dual-energy X-ray absorptiometry; FFM, fat-free mass; MRI, magnetic resonance imaging; RCT, randomized controlled trial; CI, confidence interval; ETD, estimated treatment difference; MD, mean difference; IIH, idiopathic intracranial hypertension; T2D, type 2 diabetes; SMI, skeletal muscle index; LBM, lean body mass; ANCOVA, analysis of covariance; MRS, magnetic resonance spectroscopy; CV, cardiovascular; VLCD, very-low-calorie diet; PCOS, polycystic ovarian syndrome; NAFLD, non-alcoholic fatty liver disease; MASLD, metabolic dysfunction-associated steatotic liver disease; CKD, chronic kidney disease

Number	Study/trial	Year	Registration	Population	Intervention	Comparator	Duration (week)	Body-composition modality	Eligible lean/body-composition outcome	Role in the review	Analytic role
1	STEP 1 DXA substudy: Wilding et al. (2021) [10] and Wilding et al. (2021) [11]	2021	STEP 1/NCT03548935	Adults with overweight/obesity without diabetes	Semaglutide 2.4 mg	Placebo	68	DXA	Total lean body mass by DXA. Absolute kilogram change directly reported; registry lean body mass (%) is proportion of body weight, not percent change in absolute lean mass	Primary-window DXA evidence; absolute lean-mass estimate available; registered percentage-change estimand unavailable	Key secondary: core analysis
2	SURMOUNT-1 DXA substudy: Look et al., 2025 [12]	2025	SURMOUNT-1/NCT04184622	Adults with overweight/obesity without diabetes	Tirzepatide 5/10/15 mg (body-composition results pooled)	Placebo	72	DXA	DXA total lean mass: percent and absolute change, with treatment difference and 95% CI	Registered primary direct evidence; pooled tirzepatide dose node	Primary outcome: direct estimate; key secondary: core analysis
3	Healthy weight loss maintenance/S-LiTE: Lundgren et al. (2021) [13] and Sandsdal et al. (2023) [14]	2021 and 2023	S-LiTE/NCT04122716	Adults with obesity after diet-induced weight loss	Liraglutide 3.0 mg	Placebo	52	DXA	DXA lean mass; absolute change data available	Primary-window DXA sensitivity evidence; absolute lean-mass estimate available; registered percentage-change estimand unavailable	Key secondary: sensitivity analysis
4	GLORY-1: Ji et al., 2025 [15]	2025	GLORY-1	Adults with obesity/overweight	Mazdutide 4 mg, 6 mg	Placebo	48	DXA	DXA total fat and lean mass in 114 participants; exact dose-specific lean-mass estimates/variance not publicly extractable	Primary-window DXA study; dose-specific registered-outcome estimates/precision unavailable	Primary outcome: quantitative data unavailable
5	ATTAIN-1: Wharton et al., 2025 [16]	2025	ATTAIN-1	Adults with obesity/overweight without diabetes	Orforglipron multiple doses (DXA results pooled)	Placebo	72	DXA voluntary subgroup; pooled active, n = 126; placebo, n = 45	Week-72 pooled doses: total lean mass -4.5% versus placebo +0.3%; treatment-regimen estimand	Primary-window DXA study; percentage-change point estimate available; direct precision unavailable	Primary outcome: precision unavailable
6	BELIEVE: Heymsfield et al., 2026 [17]	2026	BELIEVE	Adults with obesity	Semaglutide monotherapy arm(s); bimagrumab combinations	Placebo and active combination arms	48	DXA	DXA total body lean mass; exact semaglutide dose-specific contrasts available	Registered primary direct evidence; shared placebo covariance unavailable for joint modeling	Primary outcome: dose-specific direct estimates
7	STEP UP MRI substudy: Wharton et al., 2025 [18]	2025	STEP UP	Adults with obesity	Semaglutide 7.2 mg and 2.4 mg	Placebo	72	MRI	Lean body volume	Separate-modality MRI evidence	Supplementary: regional muscle assessment
8	SYNCHRONIZE-1 MRI substudy: le Roux et al., 2026 [19]	2026	SYNCHRONIZE-1/NCT06066515	Adults with obesity	Survodutide 3.6 mg, 6 mg	Placebo	76	MRI prespecified body-composition subgroup	Lean body volume with arm estimates at week 76	48- to <80-week separate-modality MRI evidence; direct covariance unavailable	Supplementary: MRI muscle assessment
9	MOMENTUM: Aronne et al., 2024 [20]	2024	MOMENTUM phase 2 obesity trial	Adults with overweight/obesity	Pemvidutide 1.2/1.8/2.4 mg	Placebo	48	MRI body-composition substudy	Publicly reported lean-loss ratio; no verified arm-specific lean-mass mean change/variance	Separate-modality MRI evidence; quantitative lean-effect data unavailable	Supplementary: MRI muscle assessment
10	SURPASS-3 MRI: Gastaldelli et al., 2022 [21]	2022	SURPASS-3 MRI/NCT03882970	Adults with type 2 diabetes	Tirzepatide	Insulin degludec	52	MRI	Regional fat/lean/muscle measures	Supportive evidence outside principal quantitative synthesis	Supplementary: muscle quality
11	SUSTAIN 8 body-composition substudy: McCrimmon et al., 2020 [22]	2020	SUSTAIN 8/NCT03136484	Adults with type 2 diabetes	Semaglutide 1.0 mg	Canagliflozin 300 mg	52	DXA	Total lean mass direct ETD -0.78 kg (95% CI: -1.61 to 0.04)	Supportive direct DXA active-comparator evidence	Supplementary: active-comparator DXA
12	Retatrutide phase 2 T2D body-composition substudy: Coskun et al., 2025 [23]	2025	Phase 2 T2D	Adults with type 2 diabetes	Retatrutide multiple doses	Placebo; dulaglutide	36	DXA	Lean mass exploratory; exact arm-specific lean-mass values/variance not retained for principal analysis	24- to <48-week DXA study; quantitative lean-mass estimates unavailable	Supplementary: short-duration DXA; quantitative data unavailable
13	Mazdutide phase 2: Ji et al., 2023 [24]	2023	NCT04904913	Chinese adults with overweight/obesity	Mazdutide 3/4.5/6 mg	Placebo	24	DXA exploratory subset	Total fat and total lean mass absolute change shown in supplementary figure; small subset	24- to <48-week DXA study; source-level quantitative lean-mass data unavailable	Supplementary: short-duration DXA; quantitative data unavailable
14	Exenatide for idiopathic intracranial hypertension: Mitchell et al., 2023 [25]	2023	IIH:Pressure randomized placebo-controlled trial	Adult women with active IIH and obesity	Exenatide	Placebo	12	DEXA	Lean-mass change +0.40 ± 1.00 kg versus +0.29 ± 1.50 kg; derived MD +0.11 kg	12- to <24-week DXA sensitivity evidence; unadjusted change contrast	Supplementary: short-duration DXA; unadjusted direct estimate
15	Semaglutide in HIV-associated lipohypertrophy: Eckard et al., 2024 [26]	2024	NCT04019197	Adults with HIV-associated lipohypertrophy, BMI ≥ 25 kg/m², without diabetes	Semaglutide 1.0 mg weekly	Placebo	32	Whole-body DXA + CT	Total lean body mass; model-based treatment effect -2.98 kg (95% CI: -6.83 to 0.86)	24- to <48-week DXA evidence; absolute lean-mass estimate available	Supplementary: short-duration DXA direct estimate
16	CRANIOEXE: Gatta-Cherifi et al., 2024 [27]	2024	CRANIOEXE/NCT02860923	Adults with craniopharyngioma-related obesity	Exenatide twice daily	Placebo + intensive lifestyle intervention	26	Body-composition measurement planned	No extractable lean/body-composition numerical outcome in checked report	DXA lean outcome measured but numerical results unavailable	Supplementary: DXA; numerical outcome unavailable
17	LEAD-2 DXA substudy: Jendle et al., 2009 [28]	2009	LEAD-2 (distinct randomized trial)	Adults with type 2 diabetes	Liraglutide 0.6/1.2/1.8 mg (LEAD-2)	Placebo or glimepiride (LEAD-2)	26	DXA	DXA total lean body tissue mass; absolute change (kg) at week 26	Eligible 24- to <48-week DXA evidence; reported by Jendle et al. [28]	Supplementary: short-duration DXA
18	LEAD-3 DXA substudy: Jendle et al., 2009 [28]	2009	LEAD-3 (distinct randomized trial)	Adults with type 2 diabetes	Liraglutide 1.2/1.8 mg (LEAD-3)	Glimepiride (LEAD-3; no placebo arm)	52	DXA	DXA total lean body tissue mass; absolute change (kg) at week 52	Supplementary active-comparator DXA evidence; reported by Jendle et al. [28]	Supplementary: active-comparator DXA
19	Liraglutide sustained weight loss: Astrup et al., 2012 [29]	2012	20-week obesity dose-ranging RCT	Adults with obesity without T2D	Liraglutide 1.2/1.8/2.4/3.0 mg	Placebo; open-label orlistat	20	DXA body-composition substudy	DXA lean-tissue relative change with dose-specific contrasts	12- to <24-week DXA evidence; relative percentage-change contrasts available	Supplementary: short-duration DXA percentage-change estimates
20	Liraglutide visceral fat: Bouchi et al., 2017 [30]	2017	Randomized open-label trial	Adults with T2D receiving insulin	Liraglutide + background insulin	Background insulin	24	DXA/CT; skeletal muscle index	SMI and body-composition outcomes; no total-LBM primary endpoint	Skeletal muscle index/body-composition evidence outside the principal synthesis	Supplementary: skeletal-muscle-index/body-composition evidence
21	Dapagliflozin + exenatide: Lundkvist et al., 2017 [31]	2017	NCT02313220	Adults with obesity without diabetes	Dapagliflozin 10 mg + exenatide 2 mg weekly	Double placebo	24	MRI	Total lean-tissue volume difference -0.19 L (95% CI: -0.94 to 0.56)	Exploratory combination-therapy MRI lean-tissue volume evidence; outside principal DXA synthesis	Supplementary: combination-therapy MRI lean-tissue volume
22	GLP-1 + exercise: Mensberg et al., 2017 [32]	2017	16-week randomized double-blind trial	Overweight sedentary adults with T2D	Liraglutide 1.8 mg + structured exercise	Placebo + identical exercise	16	DXA	Total LBM change +0.1 ± 2.2 versus +0.7 ± 1.5 kg; ANCOVA p = 0.42	12- to <24-week DXA sensitivity evidence	Supplementary: short-duration DXA direct estimate
23	Exenatide versus insulin glargine: Yin et al., 2018 [33]	2018	NCT02325960	Overweight/obese adults with T2D	Exenatide	Insulin glargine	16	DXA	Total/regional lean tissue measured; total lean-tissue percentage difference reported	12- to <24-week supportive DXA evidence	Supplementary: active-comparator body composition
24	Liraglutide metabolic RCT: Matikainen et al., 2019 [34]	2019	NCT02765399	Adults with type 2 diabetes	Liraglutide 1.8 mg	Placebo with weight-matched dietary counselling	16	MRI + BIA	BIA fat-free mass assessed; no extractable numerical FFM values	Qualitative secondary BIA/FFM evidence	Supplementary: bioimpedance-derived body composition
25	Liraglutide ectopic fat in South Asian T2D: van Eyk et al., 2019 [35]	2019	NCT02660047	South Asian adults with T2D	Liraglutide 1.8 mg	Placebo + standard care	26	BIA + MRI/MRS	Absolute lean body mass change directly reported; LBM (%) is body-composition proportion	24- to <48-week secondary BIA evidence; absolute estimate available	Supplementary: bioimpedance-derived lean mass
26	MAGNA VICTORIA secondary analysis: Bizino et al., 2020 [36]	2020	MAGNA VICTORIA/NCT01761318	Adults with T2D, BMI > 25 kg/m²	Liraglutide 1.8 mg	Placebo + standard care	26	BIA + MRI/MRS	Lean body mass measured by BIA; exact primary-source lean endpoint unavailable	Secondary BIA evidence; quantitative lean-mass values unavailable	Supplementary: bioimpedance-derived body composition
27	Liraglutide obesity body fat: Kadouh et al., 2020 [37]	2020	NCT02647944	Adults with obesity	Liraglutide 3.0 mg	Placebo	16	DXA	Lean body mass change reported as median (IQR)	12- to <24-week DXA evidence; distribution incompatible with prespecified mean-difference synthesis	Supplementary: short-duration DXA; incompatible distribution for quantitative synthesis
28	Liraglutide visceral/ectopic fat: Neeland et al., 2021 [38]	2021	NCT03038620	Adults with overweight/obesity, high CV risk, without T2D	Liraglutide 3.0 mg	Placebo + lifestyle	40	Neck-to-knee MRI	Total body lean-tissue volume treatment difference -0.37 L (95% CI: -0.66 to -0.08)	24- to <48-week separate-modality MRI lean-tissue evidence	Supplementary: MRI-derived total lean-tissue volume
29	Beinaglutide versus metformin: Gao et al., 2022 [39]	2022	12-week randomized weight-loss trial	Overweight/obese adults without diabetes	Beinaglutide	Metformin	12	DXA + BIA	Estimated treatment difference in lean-tissue change -1.1 kg (95% CI: -2.7 to 0.5)	12- to <24-week supportive DXA evidence; absolute estimate available	Supplementary: active-comparator DXA direct estimate
30	Tirzepatide appetite/body-composition RCT: Heise et al., 2023 [40]	2023	28-week randomized study	Adults with type 2 diabetes	Tirzepatide 15 mg; semaglutide 1 mg	Placebo; active incretin comparator	28	BOD POD/fat-free mass	FFM: placebo -0.1 kg; semaglutide -0.8 kg; tirzepatide -1.6 kg	24- to <48-week separate FFM evidence	Supplementary: non-DXA fat-free mass
31	Liraglutide versus caloric restriction: Silver et al., 2023 [41]	2023	14-week randomized three-arm trial	Adults with obesity and prediabetes	Liraglutide 1.8 mg	Sitagliptin 100 mg; caloric restriction	14	DXA	Total lean mass absolute change directly reported	Short-duration supportive evidence with non-target active comparators	Supplementary: short-duration DXA
32	VLCD, semaglutide, or combination: Anyiam et al., 2024 [42]	2024	12-week randomized pilot	Adults with T2D, BMI 27-50 kg/m²	Semaglutide to 1 mg; semaglutide + VLCD	Very-low-calorie diet	12	DXA	LBM change mean ± SEM reported across arms	12- to <24-week supportive evidence outside principal synthesis	Supplementary: diet-combination body composition
33	Exenatide versus insulin glargine body-composition RCT: Bunck et al., 2010 [43]	2010	NCT00097500	Metformin-treated adults with T2D	Exenatide	Insulin glargine	52	DXA	Total lean mass; percentage change from baseline directly reported	Supportive evidence outside principal quantitative synthesis	Supplementary: active-comparator body composition
34	Liraglutide body composition in pump-treated type 1 diabetes: Schmidt et al., 2022 [44]	2022	Lira Pump secondary analysis	Overweight adults with insulin-pump-treated type 1 diabetes	Liraglutide 1.8 mg	Placebo	26	DXA	Total fat and lean body mass	24- to <48-week DXA evidence	Supplementary: short-duration DXA; precision unavailable
35	GLORY-2: Gao et al., 2026 [45]	2026	GLORY-2/NCT06164873	Chinese adults with obesity, with or without T2D	Mazdutide 9 mg	Placebo	60	DXA subset	Total lean mass absolute change: -6.3 kg versus -0.4 kg placebo	Primary-window DXA evidence; absolute estimate available for synthesis	Key secondary: core analysis
36	Liraglutide in overweight/obese type 1 diabetes: Ghanim et al., 2020 [46]	2020	26-week randomized placebo-controlled trial	Overweight/obese adults with type 1 diabetes	Liraglutide 1.8 mg	Placebo	26	DXA + MRI	Weight loss mainly fat mass; no meaningful lean-mass change; secondary-source values available	24- to <48-week DXA evidence; direct quantitative precision unavailable	Supplementary: short-duration DXA; direct precision unavailable
37	Oral semaglutide appetite/body-composition crossover RCT: Gibbons et al., 2021 [47]	2021	NCT02773381	Adults with type 2 diabetes	Oral semaglutide 14 mg	Placebo	12	BOD POD	Whole-body lean mass change -0.1 ± 1.7 kg versus +0.5 ± 1.2 kg	12- to <24-week separate-modality lean-mass evidence	Supplementary: non-DXA body composition
38	BARI-STEP: Stanley et al., 2026 [48]	2026	BARI-STEP/NCT05073835	Adults ≥1 year after gastric bypass/sleeve with suboptimal weight loss	Semaglutide 2.4 mg weekly	Placebo	68	BIA (Tanita DC-430MA S), not DXA	Lean soft tissue mass absolute change and relative lean soft tissue mass	48- to <80-week secondary BIA evidence; absolute lean-mass estimate available	Supplementary: bioimpedance-derived lean mass
39	PCOS liraglutide RCT: Frøssing et al., 2018 [49]	2018	NCT02073929	Women with PCOS and BMI > 25 kg/m² and/or insulin resistance	Liraglutide 1.8 mg daily	Placebo	26	DXA + MRI/MRS	Adjusted between-group DXA lean-mass difference -2.3 kg (95% CI: -3.5 to -1.2)	24- to <48-week DXA evidence; absolute estimate available	Supplementary: short-duration DXA; exploratory liraglutide pool
40	PCOS liraglutide 3 mg RCT: Elkind-Hirsch et al., 2022 [50]	2022	NCT03480022	Women with PCOS and obesity	Liraglutide 3.0 mg daily	Placebo	32	DXA	Completer total LBM values reported; between-group P = 0.24	24- to <48-week DXA evidence; valid direct change-score precision unavailable	Supplementary: short-duration DXA; precision pending
41	BARI-OPTIMISE: Mok et al., 2023 [51]	2023	NCT03341429	Adults with poor weight loss after bariatric surgery	Liraglutide 3.0 mg daily	Placebo	24	DXA	Adjusted treatment difference in lean mass -3.22 kg (95% CI: -4.80 to -1.64)	24- to <48-week DXA evidence; absolute estimate available	Supplementary: short-duration DXA; exploratory liraglutide pool
42	Exenatide obesity RCT: Basolo et al., 2018 [52]	2018	Trial ID not reported	Adults with obesity without diabetes	Exenatide 10 µg twice daily	Placebo	24	DXA	Body composition measured; no posttreatment numerical lean-mass data	24- to <48-week DXA study; numerical lean-mass results unavailable	Supplementary: short-duration DXA; numerical lean-mass outcome unavailable
43	Liraglutide versus metformin/gliclazide body-composition RCT: Feng et al., 2019 [53]	2019	NCT03068065	Adults with T2D and NAFLD; mean BMI of ~27.6	Liraglutide 1.8 mg	Metformin; gliclazide	24	DXA	Total lean-tissue arm values reported; change-score variance not directly reported	24- to <48-week supportive DXA evidence; insufficient variance for weighted synthesis	Supplementary: active-comparator DXA
44	TAO exenatide schizophrenia-obesity RCT: Ishøy et al., 2017 [54]	2017	NCT01794429	Adults with obesity treated with antipsychotics	Exenatide 2 mg weekly	Placebo	14	DXA	Reported muscle-mass proxy; definition unclear	12- to <24-week secondary muscle-related outcome evidence	Supplementary: muscle-related outcome
45	Liraglutide versus empagliflozin in insulin-treated T2D: Nakaguchi et al., 2020 [55]	2020	Randomized open-label parallel RCT	Adults with T2D on stable insulin	Liraglutide 0.9 mg/day + insulin	Empagliflozin 10 mg/day + insulin	24	DXA subset	Lean-tissue mass -0.5 ± 0.3 versus -0.6 ± 0.4 kg; P = 0.80	Supportive DXA evidence outside principal quantitative synthesis	Supplementary: active-comparator DXA direct estimate
46	SURPASS J-mono body-composition substudy: Yabe et al., 2023 [8]	2023	SURPASS J-mono/NCT03861052	Japanese adults with T2D; BMI ≥ 23 kg/m²	Tirzepatide 5/10/15 mg weekly	Dulaglutide 0.75 mg weekly	52	BIA (InBody770)	Week-52 LBM change: tirzepatide -1.2/-1.9/-2.3 kg; dulaglutide -0.3 kg	Secondary BIA active-comparator evidence	Supplementary: bioimpedance-derived lean mass
47	MASLD liraglutide versus matched lifestyle: Moolla et al., 2025 [56]	2025	EudraCT 2016-002045-36	Adults with MASLD without T2D; obesity	Liraglutide	Matched calorie-deficit lifestyle	12	DXA	Arm-level total lean mass values reported; direct contrast variance unavailable	Supportive DXA evidence; arm-level values verified	Supplementary: DXA and lifestyle comparison
48	SMART prespecified body-composition analysis: Heerspink et al., 2026 [57]	2026	SMART/NCT04889183	Adults with CKD, BMI ≥ 27 kg/m², without diabetes	Semaglutide 2.4 mg weekly	Matched placebo	24	Bioimpedance spectroscopy	Direct week-24 LBM treatment effect -2.5 kg (95% CI: -6.6 to 1.6)	24- to <48-week secondary BIA/BIS evidence; direct contrast available	Supplementary: bioimpedance-derived body composition
49	Semaglutide lean-mass pilot in older adults with obesity: Ghanim et al. (2024) [58] and ClinicalTrials.gov [59]	2024	NCT05302596	Adults age ≥65 with obesity, no diabetes	Semaglutide up to 1.0 mg + standard lifestyle	Standard lifestyle/exercise only	16	DXA	Fat-free mass measured; arm-level changes available but direct randomized precision incomplete	12- to <24-week DXA evidence; direct precision unavailable	Supplementary: short-duration DXA; direct precision unavailable
50	EXPEND energy-expenditure RCT: Ravussin et al., 2025 [60]	2025	EXPEND/NCT04081337	Adults with obesity without diabetes	Tirzepatide	Placebo	18	DXA	DXA fat-free mass at week 18. The registry reports an adjusted between-group FFM change and 95% CI; source-level consistency and estimand require adjudication before any new quantitative synthesis	12- to <24-week supplementary DXA evidence; not part of registered 48- to <80-week primary or core forest plots. Existing locked analyses remain unchanged	Supplementary: short-duration DXA; not quantitatively synthesized
51	Semaglutide aging/body-composition pilot (NCT05786521): ClinicalTrials.gov [61]	2025	NCT05786521	Adults ≥65 years with overweight/obesity and prediabetes or type 2 diabetes	Semaglutide up to 1 mg weekly + lifestyle counseling	Lifestyle counseling alone	20	DXA	Lean body mass measured; registry result available; small pilot sample	12- to <24-week secondary/sensitivity DXA evidence; not eligible for 48- to <80-week core forest plots	Supplementary: short-duration DXA; not included in quantitative synthesis
52	RESETTLE: ClinicalTrials.gov, 2026 [62]	2026	RESETTLE/NCT05574439	Young adults with early-onset obesity	Semaglutide	Not specified in current audit source	68	DXA	Fat-free mass; randomized week-68 analyzable result unavailable at analysis lock	Registry-only included trial family; outcome unavailable for quantitative analysis	Supplementary: registry evidence; results unavailable

The registered primary outcome was the between-group difference in percentage change from baseline in total DXA lean body mass at 48 to <80 weeks.

The available direct contrasts for the registered primary outcome are summarized in Table 3.

**Table 3:** Candidate randomized contrasts for the registered primary DXA percentage-change outcome “Weighted direct primary evidence” indicates that both the registered effect estimate and its uncertainty were available for quantitative synthesis. “Not weighted” indicates that the contrast could not enter weighted quantitative synthesis because the required effect estimate and/or precision was unavailable. The two BELIEVE semaglutide contrasts share the same placebo group and were therefore not treated as independent observations in a joint dose-specific model DXA, dual-energy X-ray absorptiometry; CI, confidence interval; pp, percentage points; SE, standard error

Trial/study	Week	Active node	Arm percent change	Direct effect, pp (95% CI)	Status	Critical note
SURMOUNT-1 DXA substudy: Look et al., 2025 [12]	72	Tirzepatide pooled 5/10/15 mg	-10.9 versus -2.6	-8.3 (-10.6 to -6.1)	Weighted direct primary evidence	Pooled-dose efficacy estimand; direct precision reported
BELIEVE: Heymsfield et al., 2026 [17]	48	Semaglutide 1.0 mg	-4.7 versus -0.85	-3.81 (-6.57 to -1.06)	Weighted direct primary evidence	Exact treatment-regimen contrast supplied by author; shares placebo with 2.4 mg arm
BELIEVE: Heymsfield et al., 2026 [17]	48	Semaglutide 2.4 mg	-6.9 versus -0.85	-6.06 (-8.79 to -3.33)	Weighted direct primary evidence	Exact treatment-regimen contrast supplied by author; shares placebo with 1.0 mg arm
ATTAIN-1: Wharton et al., 2025 [16]	72	Orforglipron pooled doses	-4.5 versus +0.3	-4.8; precision unavailable	Not weighted	Pooled mean and n verified; exact lean-mass CI/SE unavailable
STEP 1 DXA substudy: Wilding et al. (2021) [10] and Wilding et al. (2021) [11]	68	Semaglutide 2.4 mg	Active arm -9.7%; placebo relative-percentage not recovered	Not calculable	Not weighted	Author-supplied supplement confirms DXA subgroup but not the missing registered relative-percentage contrast/precision
Healthy weight loss maintenance/S-LiTE: Lundgren et al. (2021) [13] and Sandsdal et al. (2023) [14]	52	Liraglutide 3 mg	Not recovered	Not calculable	Not weighted	Absolute per-protocol values recovered; registered relative-percentage contrast unavailable
GLORY-1: Ji et al., 2025 [15]	48	Mazdutide 4/6 mg	Dose-specific values/variance not recovered	Not calculable	Not weighted	DXA substudy confirmed; dose-specific registered outcome not publicly extractable
GLORY-2: Gao et al., 2026 [45]	60	Mazdutide 9 mg	No registered relative-percentage lean endpoint located	Not calculable	Not weighted	Absolute-kilogram endpoint available; registered relative-percentage endpoint not reported

Note that the three quantitatively usable direct contrasts originated from two independent randomized trials. The two BELIEVE dose-specific comparisons shared a placebo group and were therefore statistically correlated. Because their joint covariance was unavailable, they were not entered simultaneously as independent contrasts in the prespecified dose-specific network model. The direct effects are reported individually; a joint primary network meta-analysis was not fitted.

Primary-Outcome Findings

Three randomized contrasts had sufficient numerical information for the direct quantitative evaluation of the registered primary outcome. In SURMOUNT-1, pooled tirzepatide 5, 10, and 15 mg treatment produced a greater reduction in total DXA lean body mass than placebo at week 72, with a between-group difference of -8.3 percentage points (95% CI: -10.6 to -6.1). Author-supplied BELIEVE data provided two additional treatment-regimen contrasts at week 48: semaglutide 1.0 mg versus placebo, -3.81 percentage points (95% CI: -6.57 to -1.06), and semaglutide 2.4 mg versus placebo, -6.06 percentage points (95% CI: -8.79 to -3.33).

All three contrasts indicated the greater loss of total lean body mass with active treatment than with placebo; however, the two BELIEVE comparisons shared the same placebo group. Their covariance was unavailable; consequently, they were not treated as independent observations in a single joint dose-specific network model. The prespecified primary NMA therefore could not be estimated as a conventional independent-contrast network.

Dose-Specific Placebo-Anchored Sensitivity Analyses

Each BELIEVE contrast was instead combined separately with the independent SURMOUNT-1 contrast. The indirect comparison of tirzepatide to semaglutide 1.0 mg was -4.49 percentage points (95% CI: -8.05 to -0.93), whereas the indirect comparison of tirzepatide to semaglutide 2.4 mg was -2.24 percentage points (95% CI: -5.78 to 1.30). Negative values indicate a greater reduction in lean body mass with tirzepatide. Each individual CI is valid under the assumption that the contributing BELIEVE and SURMOUNT-1 trial estimates are independent across trials; the two sensitivity estimates should not themselves be treated as independent of one another because the underlying BELIEVE contrasts share placebo. Both estimates remain very-low-confidence cross-trial comparisons and were not used to infer superiority or rank treatments.

Key Secondary Absolute DXA Change: 48 to <80 Weeks

The key secondary absolute DXA analysis included three core direct placebo-controlled contrasts: semaglutide 2.4 mg in STEP 1, pooled tirzepatide in SURMOUNT-1, and mazdutide 9 mg in GLORY-2. S-LiTE provided additional sensitivity evidence. Core and sensitivity absolute DXA effects are summarized in Table 4.

**Table 4:** Key secondary absolute DXA lean-body-mass effects at 48 to <80 weeks Negative MD values indicate greater loss of total lean body mass with active treatment than with placebo. STEP 1, SURMOUNT-1, and GLORY-2 constitute the core key secondary dataset. S-LiTE was retained as sensitivity evidence because the week-52 analysis was per protocol and direct contrast precision was derived from reported arm confidence intervals CI, confidence interval; DXA, dual-energy X-ray absorptiometry; MD, mean difference; SE, standard error

Trial	Treatment versus placebo	Week	MD, kg	SE	95% CI	Evidence status
STEP 1: Wilding et al. (2021) [10] and Wilding et al. (2021) [11]	Semaglutide 2.4 mg	68	-3.430	0.666	-4.74 to -2.13	Core key secondary evidence: reported adjusted direct contrast
SURMOUNT-1: Look et al., 2025 [12]	Tirzepatide pooled	72	-4.400	0.612	-5.60 to -3.20	Core key secondary evidence: reported direct contrast
GLORY-2: Gao et al., 2026 [45]	Mazdutide 9 mg	60	-5.900	0.705	-7.28 to -4.52	Core key secondary evidence: exact derivation from reported arm-level SDs and sample sizes
S-LiTE: Lundgren et al. (2021) [13] and Sandsdal et al. [14]	Liraglutide 3 mg	52	-2.900	0.776	-4.42 to -1.38	Sensitivity evidence: per protocol; direct precision derived from arm CIs

Key Secondary Absolute DXA Findings

Across the core key secondary dataset, all three placebo-controlled contrasts showed greater absolute loss of total DXA lean body mass with active treatment than with placebo: -3.43 kg (95% CI: -4.74 to -2.13) for semaglutide 2.4 mg in STEP 1 [10,11], -4.40 kg (95% CI: -5.60 to -3.20) for pooled tirzepatide in SURMOUNT-1 [12], and -5.90 kg (95% CI: -7.28 to -4.52) for mazdutide 9 mg in GLORY-2 [45]. STEP 1 and SURMOUNT-1 provided direct randomized treatment contrasts, whereas the GLORY-2 effect was an unadjusted between-group difference derived from reported arm-level change data in a selected DXA subgroup. These differences in analysis population and estimand were carried forward into the transitivity and certainty assessments. These body-composition estimates should not be interpreted as direct measurements of skeletal-muscle function.

The placebo-centered evidence structure contained direct comparisons for each active treatment but no direct active-versus-active trials and no closed loops. Exploratory indirect comparisons were therefore derived from the independent placebo-controlled effects. These comparisons were not used for treatment ranking and should be interpreted cautiously because they depend on cross-trial transitivity rather than direct randomized comparison between active treatments.

Trials with usable absolute DXA lean-mass effects in kilograms and valid uncertainty were included in Figure 2, whereas studies with different outcomes, modalities, follow-up windows, or insufficient quantitative data were not displayed.

**Figure 2:**
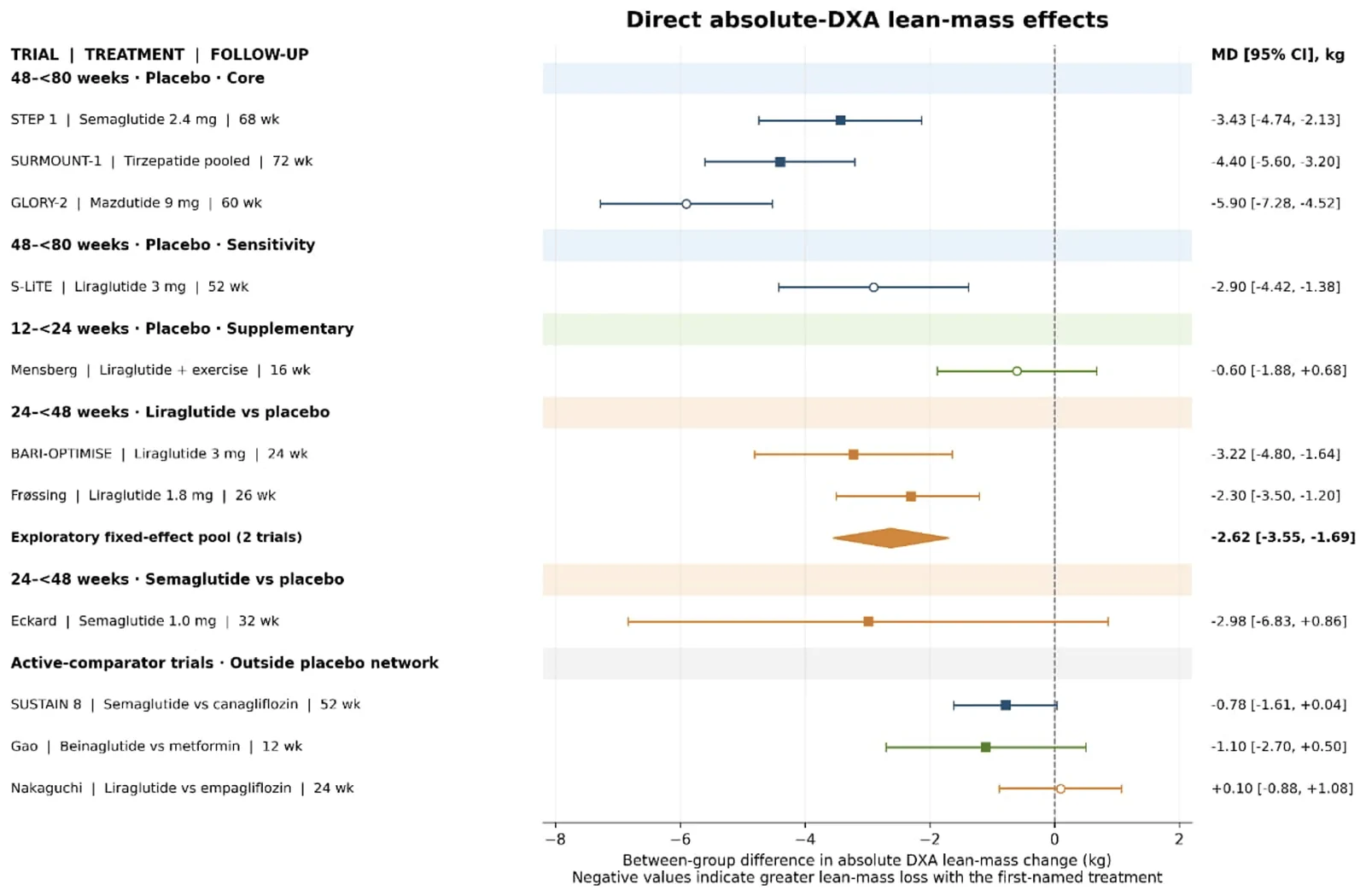
Forest plot of direct absolute DXA lean-body-mass effects Forest plot of direct treatment effects on absolute DXA lean body mass. The displayed studies are STEP 1 (Wilding et al. [10] and Wilding et al. [11]), SURMOUNT-1 (Look et al. [12]), S-LiTE (Lundgren et al. [13] and Sandsdal et al. [14]), GLORY-2 (Gao et al. [45]), Frøssing et al. [49], BARI-OPTIMISE (Mok et al. [51]), Eckard et al. [26], and IIH:Pressure (Mitchell et al. [25]). Contrasts are grouped by follow-up window and comparator type. The exploratory fixed-effect pooled estimate is shown only for the two liraglutide-versus-placebo trials at 24 to <48 weeks, Frøssing et al. [49] and BARI-OPTIMISE [51]. Squares indicate reported contrasts, open circles indicate reconstructed or sensitivity estimates, and the diamond indicates the pooled estimate. Horizontal lines indicate 95% confidence intervals DXA, dual-energy X-ray absorptiometry; MD, mean difference; CI, confidence interval

Network Geometry and Preliminary Indirect Comparisons

The absolute DXA evidence formed a placebo-centered star with semaglutide 2.4 mg, pooled tirzepatide, and mazdutide 9 mg; liraglutide 3 mg from S-LiTE was included only in sensitivity analysis [13,14]. There were no closed loops and no direct active-treatment comparisons. Heterogeneity and incoherence therefore could not be estimated empirically, and the active-treatment comparisons are exploratory cross-trial contrasts rather than treatment rankings. The network geometry is shown in Figure 3.

**Figure 3:**
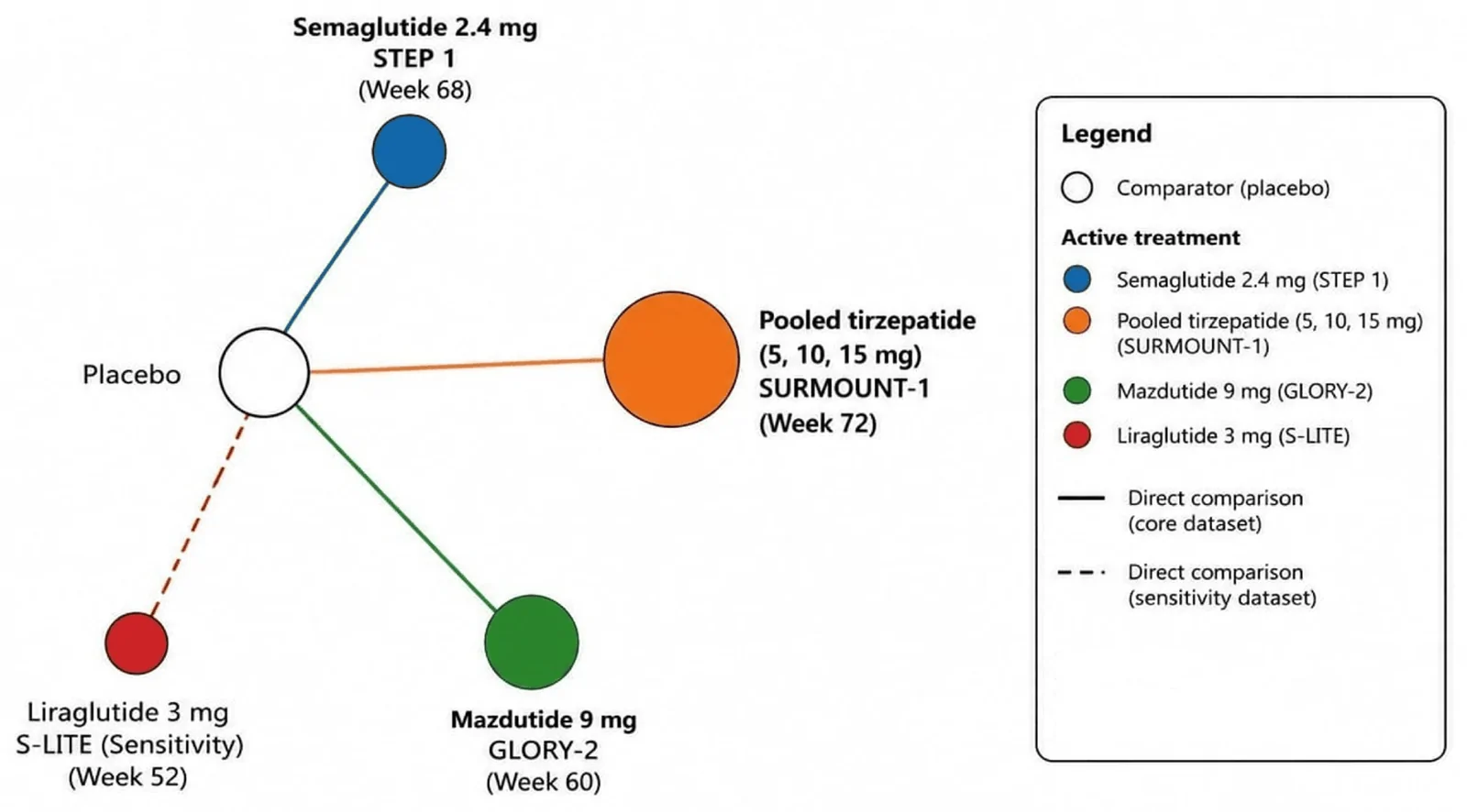
Network geometry of absolute DXA lean body mass change at 48 to <80 weeks Network geometry for the feasible absolute DXA lean-body-mass analysis at 48 to <80 weeks. Solid edges represent the core placebo-controlled contrasts from STEP 1 [10,11], SURMOUNT-1 [12], and GLORY-2 [45]; the dashed edge represents the S-LiTE sensitivity analysis [13,14] DXA: dual-energy X-ray absorptiometry

The network was sparse and placebo-centered, with no closed loops or direct active-treatment comparisons. The forest plots therefore display only analysis-eligible contrasts with compatible outcome definitions, measurement modalities, follow-up windows, and sufficiently reported treatment-effect uncertainty. Other studies in Table 2 were not included in these plots because they assessed non-equivalent body-composition outcomes or modalities, fell outside the relevant analysis window, lacked a directly extractable treatment effect or valid uncertainty estimate, or contributed only supplementary evidence. Accordingly, the forest plots represent the quantitative analytic subset rather than all trials in the broader evidence map.

Exploratory Indirect Comparisons

Using placebo as the common comparator, the exploratory indirect mean difference for semaglutide 2.4 mg versus pooled tirzepatide was +0.97 kg (95% CI: -0.80 to +2.74), while mazdutide 9 mg versus pooled tirzepatide was -1.50 kg (95% CI: -3.33 to +0.33), and mazdutide 9 mg versus semaglutide 2.4 mg was -2.47 kg (95% CI: -4.37 to -0.57). The statistical significance of any single indirect contrast was not used to infer comparative treatment superiority because each active-treatment edge was informed by one trial and transitivity could not be validated empirically.

Shorter-Duration and Separate-Modality Evidence

In the 24- to <48-week DXA window, liraglutide was represented by Frøssing et al. [49] and BARI-OPTIMISE [51]. An exploratory fixed-effect agent-level pool gave a mean difference of -2.62 kg (95% CI: -3.55 to -1.69; k = 2; I² = 0%). The I² value of 0% should not be interpreted as evidence of clinical homogeneity because populations, doses, and prior weight-loss contexts differed materially [49,51]. The fixed-effect calculation is retained as a descriptive sensitivity analysis; k = 2 is insufficient for the stable estimation of between-study variance.

In a 32-week randomized trial in people with HIV-associated lipohypertrophy, semaglutide 1.0 mg had a model-based absolute total lean-body-mass treatment effect of -2.98 kg (95% CI: -6.83 to 0.86) versus placebo [26]. At 12 weeks, the randomized exenatide IIH:Pressure DXA substudy yielded an unadjusted change-score mean difference of +0.11 kg (standard error {SE}: 0.651; 95% CI: -1.17 to 1.39) and was retained as imprecise short-duration sensitivity evidence [25]. Separate-modality evidence was not pooled with DXA. In SMART, bioimpedance spectroscopy (BIS) showed a direct lean-body-mass treatment effect of -2.5 kg (95% CI: -6.6 to 1.6) for semaglutide 2.4 mg versus placebo at 24 weeks [57].

In the SURPASS J-mono body-composition substudy, author-supplied bioelectrical impedance analysis (BIA) data showed adjusted tirzepatide-versus-dulaglutide differences of -0.9 kg (95% CI: -2.8 to 0.9) for 5 mg, -1.7 kg (95% CI: -3.4 to 0.1) for 10 mg, and -2.0 kg (95% CI: -3.8 to -0.2) for 15 mg. Because the three contrasts shared the same comparator and their joint covariance was unavailable, they were reported separately and were not pooled as independent estimates [8]. SUSTAIN 8 provided supportive direct active-comparator DXA evidence but did not bridge the prespecified incretin network [22].

Risk of Bias

All 52 included trial families were considered for outcome-level RoB 2 assessment. Forty-five had sufficiently reported eligible outcomes to permit domain-level judgments: 40 were judged to have some concerns, and five were judged at high risk of bias. Seven trial families were classified as not assessable because an eligible lean- or muscle-related body-composition outcome was identified, but no sufficiently reported outcome-level result was available to permit a defensible RoB 2 judgment. The principal sources of concern were incomplete outcome data, selection into body-composition substudies or evaluable analysis sets, open-label or incompletely masked treatment allocation in some trials, and selective or secondary reporting of lean-mass outcomes. The complete study-level traffic-light assessment is shown in Figure 4.

**Figure 4:**
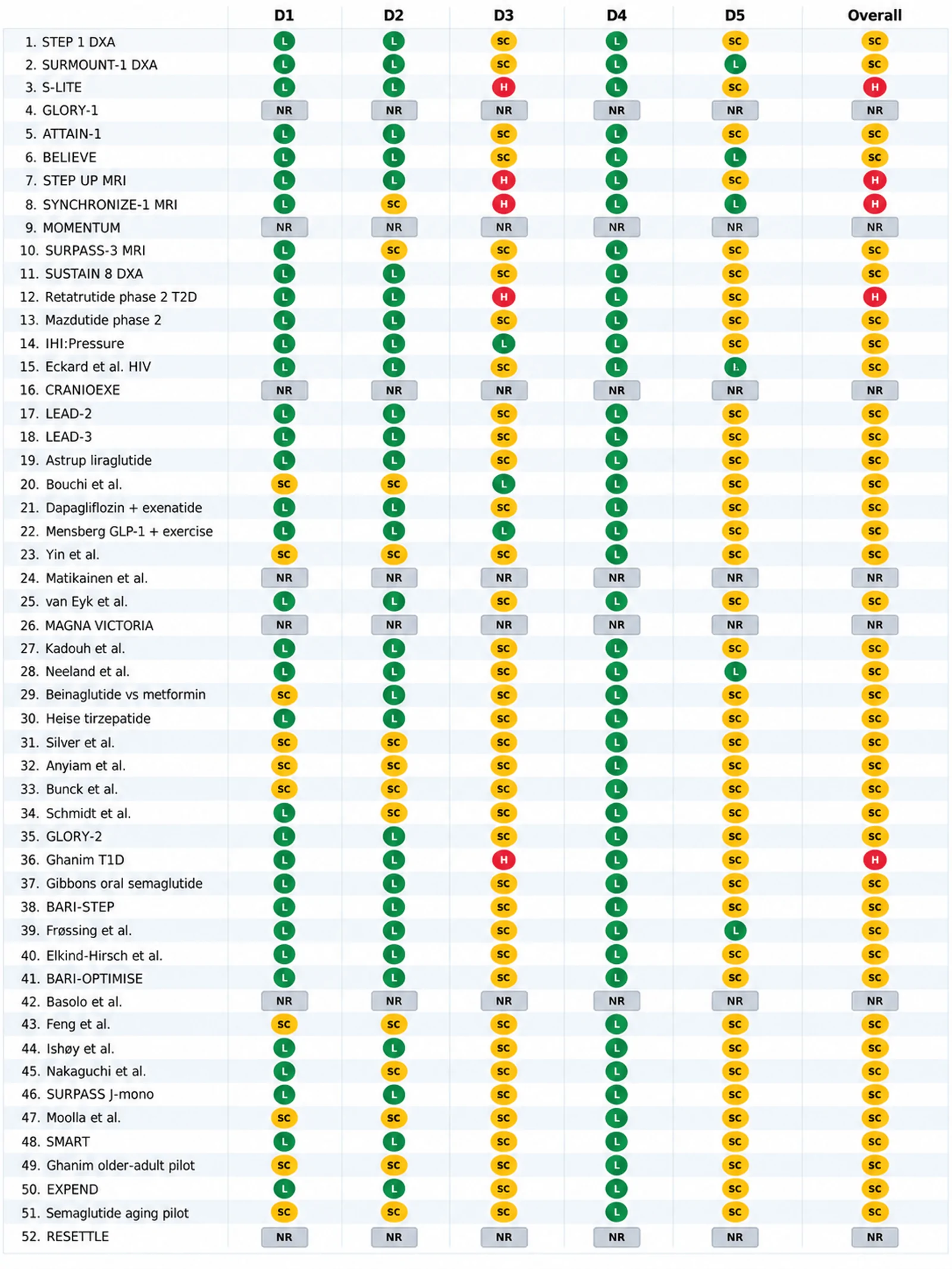
Outcome-level Cochrane Risk of Bias 2 (RoB 2) traffic-light assessment across included trial families D1, randomization process; D2, deviations from intended interventions; D3, missing outcome data; D4, measurement of the outcome; D5, selection of the reported result. L, low risk; SC, some concerns; H, high risk; NR, not assessable. For NR studies, an eligible lean- or muscle-related body-composition outcome was identified, but no sufficiently reported outcome-level result was available to permit a defensible RoB 2 judgment. Risk-of-bias judgments were based on the corresponding trial reports and registry records [8,10-62] MRI, magnetic resonance imaging; DXA, dual-energy X-ray absorptiometry; T2D, type 2 diabetes; T1D, type 1 diabetes

Transitivity Audit

Potential effect modifiers included population context, diabetes status, baseline BMI and lean mass, age and sex distribution, dose and dose pooling, duration, measurement modality, pre-randomization weight-loss interventions, structured exercise/protein co-intervention, selection into body-composition substudies, and estimand. STEP 1 and SURMOUNT-1 were the most comparable conventional obesity trials, although their body-composition estimands were not identical. GLORY-2 enrolled Chinese adults and relied on a selected DXA subgroup. S-LiTE differed more substantially because participants completed an eight-week low-calorie diet before randomization to weight-maintenance strategies. Shorter-duration studies were more heterogeneous still. We therefore did not combine studies solely because they shared a placebo comparator.

CINeMA and Confidence Assessment

The full registered primary dose-specific network did not receive a formal CINeMA network grade because both BELIEVE dose contrasts could not be entered simultaneously without their shared-control covariance. The direct BELIEVE and SURMOUNT-1 contrasts were each usable direct evidence; the two placebo-anchored semaglutide-versus-tirzepatide sensitivity comparisons were graded very low confidence because they were purely indirect and depended on cross-trial exchangeability. In the key secondary absolute-DXA analysis, confidence was low for semaglutide 2.4 mg versus placebo and pooled tirzepatide versus placebo and very low for mazdutide 9 mg versus placebo and all indirect active-treatment comparisons.

Additional Evidence and Analysis Status

Additional evidence and unresolved numerical fields were reviewed individually. Values that remained unavailable or non-equivalent were retained as data-blocked rather than inferred from ambiguous uncertainty measures. The final analysis incorporated two quantitative author-data amendments: SURPASS J-mono for secondary BIA/BIS outcomes [8] and BELIEVE for the registered primary relative percentage DXA outcome [17]. No definitive treatment ranking was produced, and unresolved data limitations did not alter the registered estimand.

Discussion

Principal Findings

Three findings are central to this review. First, direct randomized evidence for the registered percentage-change DXA outcome is available for pooled tirzepatide and two semaglutide doses, but the full dose-specific network remains constrained by shared-control covariance and estimand differences. Second, the key secondary absolute DXA evidence consistently indicates greater total lean-body-mass loss with semaglutide 2.4 mg, pooled tirzepatide, and mazdutide 9 mg than with placebo. Third, the evidence base remains too sparse and heterogeneous to support a reliable comparative treatment ranking. These findings separate what is directly estimable from what remains structurally uncertain.

Numerically, mazdutide had the most negative point estimate among the three core absolute DXA placebo comparisons, followed by pooled tirzepatide and semaglutide 2.4 mg; however, these cross-trial differences should not be interpreted as a treatment ranking. The mazdutide estimate was derived from arm-level change data in a selected DXA subgroup, whereas the STEP 1 and SURMOUNT-1 effects were differently modelled randomized treatment contrasts, adding estimand and analysis-population indirectness. The placebo-anchored sensitivity comparison between semaglutide 1.0 mg and pooled tirzepatide excluded zero, whereas the semaglutide 2.4 mg comparison did not. Neither establishes superiority because both are cross-trial indirect estimates, the BELIEVE and SURMOUNT-1 estimands are not identical, and there is no closed-loop or direct active-versus-active trial. The same caution applies to the exploratory absolute DXA comparisons; point estimates are hypothesis-generating rather than a treatment hierarchy.

Clinical Interpretation

The loss of DXA-derived lean body mass is common during substantial weight reduction, but its clinical meaning should not be inferred from the DXA value alone. DXA lean mass includes skeletal muscle together with water and other non-fat soft tissues; it does not directly measure anatomical skeletal-muscle mass, muscle quality, intramuscular fat, strength, or physical performance. Accordingly, treatment-associated reduction in DXA lean mass is not, by itself, evidence of sarcopenia or clinically harmful muscle loss [3-5].

Absolute lean-mass change also needs to be interpreted in the context of the magnitude and composition of total weight loss. A treatment producing greater overall and fat-mass reduction may produce a larger absolute decline in lean mass without necessarily causing greater impairment in muscle strength or function. Conversely, lean mass as a proportion of body weight can increase while absolute lean tissue falls, which is why ratio-based outcomes were not accepted as substitutes for absolute or relative change in total lean mass. Clinical relevance is likely to depend on age, baseline frailty or sarcopenic risk, protein intake, resistance exercise, comorbidity, and functional reserve; these factors were inconsistently reported across the included trials.

Comparison With Broader Evidence Syntheses and Implications of Incomplete Database Coverage

The incomplete CENTRAL record-level screen and the lack of native Embase, Scopus, and Web of Science searching create a real possibility of retrieval bias; however, comparison with broader recent syntheses helps estimate its likely magnitude. Eisa and Barood searched PubMed, Embase, CENTRAL, and Web of Science and included 20 randomized trials [4]. Their eligibility was broader than ours, including lifestyle interventions, DXA and MRI, absolute lean-mass change, and the proportion of total weight loss attributable to lean mass. They nevertheless reached the same qualitative conclusion: substantial weight reduction with incretin therapy is accompanied by measurable lean-mass reduction. Their higher study count therefore cannot be interpreted as 20 protocol-eligible trials that were missed by our review.

Rakhsha et al. searched PubMed, Web of Science, and Scopus and included 41 trials, but their network encompassed multiple antidiabetic drug classes rather than 41 incretin trials [63]. Their random-effects NMA found a tirzepatide lean-body-mass effect of -4.40 kg versus placebo (95% CI: -7.58 to -1.22), exactly matching the point estimate of our direct SURMOUNT-1 analysis (-4.40 kg; 95% CI: -5.60 to -3.20), despite different network structure and eligibility. This numerical concordance is supportive but does not prove the absence of retrieval bias.

Taken together, these broader searches reduce concern that incomplete CENTRAL/Embase/Scopus/Web of Science coverage would reverse the principal qualitative conclusion that incretin-associated weight loss is accompanied by measurable lean-mass reduction. The residual impact is more plausibly on completeness, treatment-specific precision, less-represented agents, and network geometry. In particular, a missed active-active trial or an additional study on an existing edge could materially alter NMA connectivity, heterogeneity assessment, or comparative precision even if the overall direction of lean-mass change remained unchanged. These external comparisons suggest that incomplete retrieval is less likely to alter the overall direction of the principal finding than to affect study completeness, treatment-specific precision, and network geometry; however, the magnitude of retrieval bias cannot be quantified from the available data.

Feasibility of the Prespecified Primary NMA

The prespecified analysis required compatible randomized contrasts for the same primary estimand, measurement modality, and time window, together with valid estimates of uncertainty. The BELIEVE author response resolved direct precision for two semaglutide doses but did not provide the covariance required to account for their shared placebo group. Treating these correlated contrasts as independent would underestimate uncertainty. Consequently, the two BELIEVE dose-specific contrasts were evaluated separately with the independent SURMOUNT-1 placebo comparison rather than entered simultaneously into a single dose-specific model. This limitation reflects the structure and reporting of available evidence rather than the absence of relevant randomized data. Preserving the prespecified outcome definition and statistical assumptions was preferable to constructing a more complete network through unverified covariance assumptions, the substitution of non-equivalent body-composition outcomes, or pooling across incompatible estimands.

Strengths

Strengths include the strict separation of percentage change, absolute kilograms, lean-mass proportion, and other body-composition constructs; a primary-source hierarchy incorporating official source data and supplements; trial-family mapping to reduce double counting across parent trials, substudies, and follow-up publications; outcome-level RoB 2 with the explicit consideration of substudy selection, missingness, and estimand differences; the conservative handling of shared controls; reproducible statistical formulas and computational verification; and the avoidance of treatment ranking when the evidence structure does not support reliable heterogeneity or incoherence assessment [7].

Limitations

The most important review-level limitation was incomplete access to some bibliographic sources. PubMed and ClinicalTrials.gov provided native record-level exports and form the auditable record-level screening flow. CENTRAL was searched and yielded 8,250 trial records, but those records could not be individually screened because reproducible citation export was unavailable. Embase, Scopus, and Web of Science were not searched because institutional/subscription access was unavailable, and the WHO ICTRP did not provide a reproducible native export during the final search window. Consequently, the evidence base cannot be claimed to be fully comprehensive. Cross-checking against broader recent systematic reviews with access to these databases produced directionally concordant conclusions and reduces concern about the reversal of the main finding, but it does not eliminate possible effects on completeness, precision, and network geometry [4,63].

For the registered primary outcome, three direct contrasts were quantitatively usable after the BELIEVE amendment, yet the two BELIEVE contrasts shared a placebo group and their covariance was unavailable; the full dose-specific NMA was therefore not estimated. Several other primary-window studies remained blocked by missing or non-equivalent outcomes or incomplete precision. The 48- to <80-week absolute DXA evidence was also sparse, with one study per core edge and no closed loops, precluding the empirical assessment of incoherence or edge-level heterogeneity. In addition, the GLORY-2 mazdutide contrast was derived from arm-level change data in a selected DXA subgroup rather than from the same type of adjusted treatment estimand available for all other core edges. These limitations prevent definitive comparative treatment ranking.

Several body-composition analyses were based on selected substudies or per-protocol/complete-case populations. Restriction to DXA-capable sites, the availability of paired scans, and the exclusion of participants with missing follow-up measurements may reduce generalizability and can introduce selection bias if measurement availability is related to treatment response, tolerability, achieved weight loss, or unobserved lean-mass outcomes. Screening and data extraction were cross-checked, and key quantitative fields were independently verified, while the principal RoB 2 set underwent second review and adjudication; however, a fully independent duplicate screening pass for every screened record is not documented in the retained audit trail. Finally, total DXA lean body mass remains an imperfect surrogate for the clinically meaningful preservation of skeletal muscle and function.

Implications for Research

Future trials should report randomized arm-level baseline and follow-up values for total and appendicular lean mass together with exact between-group contrasts, uncertainty estimates, analysis population, and missing-data methods. Functional outcomes such as muscle strength and physical performance should be incorporated more consistently. For multi-arm trials, source data should allow the reconstruction of covariance between contrasts sharing a common control. Body-composition substudies should report how participants were selected, the proportion undergoing baseline and follow-up scans, and why measurements are missing so that treatment effects can be interpreted without unverifiable assumptions.

## Conclusions

Randomized trials show that incretin-based pharmacological weight loss can be accompanied by reductions in DXA-derived total lean body mass. These measurements should not be interpreted as direct evidence of clinically harmful skeletal-muscle loss, sarcopenia, or impaired function. Direct relative-percentage DXA effects were available for pooled tirzepatide and two semaglutide doses, but the full prespecified dose-specific network could not be estimated without making an unsupported independence assumption for the shared-placebo BELIEVE contrasts. Separate indirect sensitivity analyses were of very low confidence and do not establish comparative treatment superiority or support treatment ranking. The absolute DXA evidence supports greater lean-mass loss with several active treatments than with placebo, while separate BIA/BIS findings provide additional lower-certainty information. Concordant findings from broader reviews with Embase, CENTRAL, Web of Science, and/or Scopus coverage reduce concern that incomplete database access would reverse the principal qualitative conclusion, but they do not eliminate uncertainty about study completeness, treatment-specific precision, or network geometry. A more consistent reporting of total and appendicular lean mass, exact randomized contrasts and covariance for multi-arm studies, and the routine assessment of strength and physical function are needed before the comparative effects of incretin therapies on muscle health can be defined with confidence.
